# Integrative oncology for young adults with cancer: A prospective pragmatic controlled trial

**DOI:** 10.1007/s12032-026-03312-8

**Published:** 2026-06-23

**Authors:** Eran Ben-Arye, Shahar Samuel, Ilit Turgeman, Noah Samuels, Nili Stein, Orit Gressel

**Affiliations:** 1 Ruth and Bruce Rappaport Faculty of Medicine, Technion-Israel Institute of Technology, Haifa, Israel; 2 Integrative Oncology Program, The Oncology Service, Lin, Zebulun, and Carmel Medical Centers, Clalit Health Services, Haifa, Israel; 3 The Oncology Service, Lin and Zebulun Medical Centers, Clalit Health Services, Haifa, Israel; 4 Center for Integrative Complementary Medicine, Faculty of Medicine, Shaare Zedek Medical Center, Hebrew University of Jerusalem, Jerusalem, Israel; 5 Department of Community Medicine and Epidemiology (N.S.), Carmel Medical Center, Haifa, Israel; 6 The Oncology Service, Lin Medical Center, 35 Rothschild St, Haifa, Israel

**Keywords:** Integrative oncology, Young adults, Quality of life, Integrative medicine, Adherence

## Abstract

Research on the impact of integrative oncology (IO) programs on quality of life (QoL) among young adults with cancer is limited. The present study explored the impact of a patient-tailored IO program for young patients undergoing active oncology care. This pragmatic, prospective and preference-controlled study examined patients aged 18–45 referred by oncology healthcare providers to an IO consultation and 6 weekly treatments addressing QoL-related concerns. High adherence to integrative care (high-AIC, vs. low-AIC) was defined as attending ≥ 4 IO sessions, with baseline and 6-week symptoms assessed using ESAS (Edmonton Symptom Assessment Scale); EORTC QLQ-C30 (European Organization for Research and Treatment of Cancer Quality of Life Questionnaire) and MYCAW (Measure Yourself Concerns and Wellbeing) tools. Of 180 patients, 137 (76%) were high-AIC, with similar baseline demographic, cancer-and QoL-related characteristics in both groups. At 6 weeks, high-AIC patients reported greater improvement on ESAS (*p* = 0.046) and MYCAW (*p* = 0.008) wellbeing; ESAS nausea (*p* = 0.027); EORTC dyspnea; and EORTC role functioning (*p* = 0.031). Significant within-group improvement was observed in older high-AIC patients (aged 40–45, *n* = 72), as opposed to no improvement in young high-AIC patients (aged 18–39, *n* = 65) on ESAS fatigue (*p* = 0.033), anxiety (*p* = 0.001), and breathing (*p* = 0.016); and wellbeing on ESAS (*p* = 0.006) and MYCAW (*p* < 0.001). Young patients with cancer highly adherent to a 6-week IO program reported significantly improved wellbeing, more significantly among older patients. Further research is needed to explore the impact of IO programs on young patients with cancer, addressing their unique psychological, social, and spiritual needs.

## Introduction

Young adults with cancer often have unmet needs, as well as limited access to oncology services which has been attributed to their unique psycho-social and quality of-life (QoL)-related concerns [1]. In 2010, the U.S. National Cancer Institute (NCI) published a position statement highlighting critical elements needed to ensure quality of care for adolescents and young adults (AYA) patients, including the promotion of adherence and access to healthcare providers (HCPs) [2]. A 2021 position paper from the European Society for Medical Oncology recommended diversifying inter-professional cooperation in AYA care [3]. Zebrack et al. reported that AYA patients are more likely to report unmet needs from services providing complementary and alternative medicine [4]. In Israel, a cross-cultural study conducted among 770 Jewish and Arab patients with cancer found that young age was associated with higher rates of complementary medicine use for cancer-related outcomes [5].

Integrative oncology (IO) is defined by the Society for Integrative Oncology as “a patient-centered, evidence-informed field of cancer care that aims to optimize health, QoL, and clinical outcomes across the cancer care continuum“ [6]. In contrast to the large body of evidence supporting the role of IO programs in addressing QoL-related needs among adult patients with cancer, there has been little research exploring their role in the treatment of AYA patients with cancer. Moreover, the effective and safe use of IO modalities recommended in the clinical practice guidelines co-published by the Society for Integrative Oncology (SIO) and the American Society of Clinical Oncology (ASCO) for patients with breast cancer [7]; cancer-related pain [8]; anxiety and depression [9]; and cancer-related fatigue [10] do not address the AYA population of patients. The present study explores the impact of a patient-tailored IO program implemented within an oncology service in Israel assessing QoL-related outcomes among young patients undergoing active oncology treatment.

## Methods

### Study design and patient population

The study was conducted within a pragmatic and prospective methodology. It was patient preference-controlled, without randomization, with recruitment, consultations and IO treatments taking place at the Oncology Service of Clalit Healthcare Services (the largest Healthcare Maintenance Organization in Israel, covering nearly half of the population in the country) at the Lin, Zebulon and Carmel medical centers in northern Israel. The IO service annually treats approximately 1,400 new patients from northern Israel, with the study centers providing the overwhelming majority of medical oncology treatments, with radiation treatments provided at other collaborating oncology centers in the region. The IO program at the three centers, established in 2008, has been providing treatments addressing QoL-related concerns within the context of supportive and palliative care at each [11].

Eligible participants were aged ≥ 18 and ≤ 45 years, and had been referred by one of their oncology HCPs to an IO consultation and treatments (from July 2009 to March 2025), this while undergoing active oncology treatment (chemotherapy, biological treatment, and/or immunotherapy) for solid cancer. The range of age chosen for study inclusion was based on the minimum age required for all patients referred to and inducted into the Oncology Service at the Clalit Healthcare Services (i.e., ≥ 18 years); the U.S. NCI definition of AYA (between ages 15 and 39 years) [12]; and the range of ages reported in cases of early-onset cancer, depending on the cancer type [13]. In order to address all age-related factors in the present study, two subgroups were defined: young patients, aged ≥ 18 and ≤ 39 years; and older patients, aged ≥ 40 and ≤ 45 years. The rationale behind the division of the study cohort to these two subgroups was based on clinical observation, shared by the teams of oncologists, nurse oncologists, psycho-oncologists, and IO providers in the Oncology Service; as well as the unique spectrum of challenges faced by oncology patients under the age of 40. These include fertility-related distress; partnership status; parenting status; dependency on caregivers (including vis-à-vis parents); disruption of education; employment and career; and significant emotional and spiritual distress related to the cancer diagnosis, which is often perceived as ‘unpredictable’; ‘unjustified’ and not in alignment with the health perception of this age group.

### Referral to the IO consultation

Oncology HCPs, including oncologists, nurse oncologists and psycho-oncologists, referred patients to an IO consultation provided by an integrative physician (IP). The referral required that the HCP provide at least one QoL-related indication such as pain, emotional distress, fatigue, or gastro-intestinal symptoms. During the IO consultation (typically lasting an hour) the IP assessed QoL-related concerns and well-being, with treatment goals co-defined with the patient and a 6-week IO treatment program then scheduled. IO treatments were provided by a multidisciplinary team of physicians, nurses, paramedical HCPs (registered dietitians, social workers, occupational therapists); and IO therapists (including traditional Chinese medicine, manual, movement, and mind-body-spirit practitioners), all of whom had undergone a 270-hour training program in integrative and supportive cancer care. Among the IO team, two practitioners had undergone additional AYA-specific training, including by an integrative physician on the faculty of the program and another physician with extensive experience in the pediatric IO setting. In addition, the national IO training syllabus included AYA-related teaching as well [14]. IO treatments were adapted, to some extent, to meet the unique needs of young adult patients, and prioritized to IO AYA-trained or experienced IO practitioners with highly tuned communication-related skills with both patients and informal caregiver.

### Patient-tailored IO treatments

The goal of the IP consultation is to co-design with the patient an IO treatment program, while taking into consideration the severity of the patient’s concerns and wellbeing; the research-based evidence on the safety and effectiveness of the planned IO treatments, in accordance with the SIO-ASCO and other clinical practice guidelines; and the patient’s expectations and health-belief models, including past experience with complementary medicine. At the end of each IO consultation the IP provides a summary letter describing the recommended IO treatment plan, to be sent to the patient’s oncologist, nurse oncologist, psycho-oncologist, and family physician. This facilitates further exploration of unmet patient needs, as perceived from the HCP’s perspective, with the ability to provide additional therapeutic suggestions to enhance the IO treatment goals and plan.

In the absence of specific published IO clinical guidelines for the treatment of AYA patients, and in light of the limited number of randomized controlled trials examining oncology young adult population [15], the present study used IO modalities as recommended in the SIO-ASCO clinical guidelines for adults in the treatment of cancer-related pain, anxiety/depression, and cancer-related fatigue, adapted to the complexity of the individual patient’s spectrum of QoL-related concerns.

Weekly patient-tailored IO treatment sessions (lasting between 30 and 45 min) included at least one of the following modalities: guidance on herbal and dietary supplement use; manual therapies (e.g., acupuncture, reflexology, manual Anthroposophic techniques); movement therapies (e.g., QiGong, Feldenkrais, Paula method); and mind-body-spirit therapies (guided imagery, music therapy, spiritual care). Conventional palliative/supportive care was provided throughout the study period, including consultations and medications provided by oncologists and palliative care specialists; nurse-provided care; and psycho-oncology interventions, among others.

### Adherence to IO treatments

During the IO consultation, the IP emphasizes the importance of adhering to weekly IO treatments for at least 6 weeks, with the goal of providing adequate relieve for both short- and long-term QoL-related concerns. Moreover, the IP tries to ensure that a continuum of weekly IO treatment be provided to patients, with ongoing assessment and constant adjustment of treatments to address the patient’s leading QoL-related concerns. IO treatments are scheduled to begin within a week of the IP consultation, with the IO team of practitioners aware of potential non-adherence to the planned regimen. Thus, at the conclusion of each session, IO practitioners make sure to schedule the next visit within a week. Patients who cancel or do not attend are contacted by telephone to assess the reasons for non-attendance, and if possible, to re-schedule within the coming days (up to a week, if possible). At 6 weeks, patients attending the IP consultation were designated to two study groups, based on their adherence to the weekly integrative care program. High adherence to integrative care (high-AIC) was defined as attending ≥ 4 treatments during the 6-week study period. Patients attending ≤ 3 sessions were designated as low-AIC.

### QoL-related outcomes

QoL-related outcomes were scored at baseline; during the initial IP visit; and at 6-weeks, using the Edmonton Symptom Assessment Scale (ESAS) [16]; the Measure Yourself Concerns and Wellbeing (MYCAW) questionnaire, which included patient narratives describing their experience at follow-up [17]; and the European Organization for Research and Treatment of Cancer Quality of Life Questionnaire (EORTC QLQ-C30) [18]. All three questionnaires (ESAS [19], MYCAW [20], and EORTC QLQ C-30 [21]) have been used as a patient-reported outcome measures in the assessment of AYA QoL-related concerns and wellbeing. The ESAS tool scores 10 symptoms over a 24-hour period, from 0 (none) to 10 (worst possible severity). The MYCAW questionnaire asks patients to list and score (from 0 to 6) their two most important concerns, as well as general wellbeing. Finally, the EORTC asks patients to score severity of their symptoms and overall health over the past 7 days. Functional EORTC scores range from 0 (worst level) to 100 (best level), and symptoms from 0 (lowest severity) and 100 (greatest severity).

### Statistical analysis

Statistical analyses were performed using IBM SPSS Statistics, version 28.0 (IBM, New York, NY). At least 45 patients were deemed necessary in each group to assess the impact of IO on study outcomes (OpenEpi program, Microsoft), for an alpha-error of 0.05 and beta-error of 0.2 (power 80%) assuming a difference of a 25% improvement on ESAS 11-point scales. Continuous variables are presented using means and standard deviations (SD) or medians and interquartile ranges (IQR); with categorical variables presented using counts and proportions. Comparisons of patient characteristics between patients with high vs. low adherence, or patients in a young vs. older age subgroup were performed using a Chi-square test for categorical variables. For continuous variables, either an independent t-test or Mann-Whitney U test were used, depending on data distribution. A two-tailed significance level of 0.05 was used for all statistical tests in the study.

Differences in ESAS, EORTC and MYCAW scores from before and after integrative treatment were assessed within each group (high AIC; low AIC; high AIC, age 18–39 years old; high AIC, age 40–45 years old) using the Wilcoxon signed-rank test. The differences between pre and post treatment scores were calculated for each patient, with between-group comparison performed using a Mann-Whitney test, for both baseline scores as well as calculated differences.

### Ethical considerations

The study protocol was approved (0024-09-CMC) by the Ethics Review Board (Helsinki Committee) at the Carmel Medical Center in Haifa, Israel, and registered at ClinicalTrials.gov (NCT01860365).

## Results

### Baseline characteristics

Of the 243 eligible patients seen by the study IP, 165 (67.9%) were high-AIC and 78 low-AIC. Table 1 presents the characteristics of 180 patients for whom both baseline and 6-week QoL assessments were available, of which 137 (76%) were designated as high-AIC and 43 as low-AIC. As shown in the table, the two groups had similar baseline demographic, cancer-, and oncology treatment-related parameters. While rates of prior use of complementary medicine in general were similar in both groups, the use of these treatments for cancer-related goals was more prevalent in the high-AIC group (*p* = 0.012).

**Table 1:** Comparison of characteristics of young adult patients with cancer, comparing those high vs. low-AIC groups

Characteristic	High AIC *n* = 137	Low-AIC *n* = 43	*P* values
*Age* Mean ± SD (median)	39.1 ± 4.7	39.0 ± 5.0	0.854
*Gender/Sex* Female	122 (89.1)	38 (88.4)	> 0.99
*Primary Language* Hebrew Arabic Russian Other	84 (61.3) 42 (30.7) 10 (7.3) 1 (0.7)	30 (69.8) 10 (23.3) 2 (4.7) 1 (2.3)	0.541
*Country of Birth* Israeli born	113 (82.5)	35 (81.4)	0.871
*Residence* Haifa & suburbs (vs. periphery)	71 (51.8)	22 (51.2)	0.940
*Education* Academic	98 (77.2)	26 (63.4)	0.082
*Occupation* Employed	79 (60.8)	13 (31.0)	< 0.001
*Income* National Average & below	79 (61.7)	36 (87.8)	0.002
*Marital status* Married	106 (79.1)	28 (66.7)	0.099
*Children* Number mean ± std. Median (IQR)	2.5 ± 1.2 3 (2; 3)	2.5 ± 1.2 2 (2; 3)	0.804
*Informal caregiver* Married/spouse	101 (79.5)	28 (68.3)	0.138
*Prior general complementary medicine use* Yes	88 (64.2)	28 (65.1)	0.916
*Cancer-related complementary medicine use:* Yes	78 (56.9)	15 (34.9)	0.012
*Primary cancer site:* Breast Gynecologic oncology Gastro-intestinal Lung Prostate Urinary	105 (76.6) 10 (8.2) 14 (10.2) 3 (2.2) 0 5 (3.6)	31 (72.1) 4(10.5) 7(16.3) 0 0 1(2.3)	0.545 0.743 0.280 > 0.99 > 0.99
*Cancer recurrence* Yes	16(11.7)	4 (9.3)	0.787
*Cancer metastasis* Yes	31 (23.1)	8 (18.6)	0.533
*Oncology treatment setting* Adjuvant Neo-adjuvant Palliative Other	71 (54.2) 35 (26.7) 0 25 (19.1)	22 (51.2) 13 (30.2) 1 (2.3) 7 (16.3)	0.391

AIC, adherence to integrative care.

### IO treatment modalities

Table 2 presents the different IO modalities provided to the study patients during the 6-weeks of treatment, with acupuncture the most frequently one used (95.6% in high-AIC; 65.1% in low-AIC patients), followed by manual-movement, herbal medicine, mind-body, and Anthroposophic medicine. Patients in the high-AIC group received a greater total number of IO modalities (mean 3.7 ± 0.96 vs. 2.6 ± 1.2, *p* < 0.001), and a significantly higher number of IO interventions during the 6-week study period (*p* < 0.001).

**Table 2:** Integrative oncology modalities used to treat young adult patients with cancer during the 6-week IO treatment program

IO modality	High AIC *n* = 137	Low-AIC *n* = 43	*P* values
Acupuncture	131 (95.6)	28(65.1)	< 0.001
Manual-movement	128 (93.4)	28(65.1)	< 0.001
Herbal medicine	116 (84.7)	33 (76.7)	0.230
Mind-body	85 (62.0)	16 (37.2)	0.004
Anthroposophic medicine	46 (33.6)	6(14.0)	0.013
*Number of IO treatment days* Mean ± SD	6.7 ± 1.9 7 (5; 8)	2.7 ± 1.7 2 (1; 4)	< 0.001
*Number of IO interventions* Mean ± SD	20.1 ± 8.6 20 (14; 25)	6.7 ± 1.9 7 (5; 8)	< 0.001
*Number of modalities practiced:* 1–2 ≥ 3	12(8.8) 125 (91.2)	19 (44.2) 24 (55.8)	< 0.001
Mean of practiced IO modalities	3.7 ± 0.96	2.6 ± 1.2	< 0.001

IO, integrative oncology

### QoL-related changes from baseline to 6 weeks

Table 3 presents baseline-to-6-week changes in QoL-related outcomes. Patients with high-AIC reported a more significant improvement in wellbeing scores at 6 weeks on ESAS (*p* = 0.046) and MYCAW (*p* = 0.008), with no between-group difference for EORTC scores (*p* = 0.265). Between-group analysis showed a more favorable response to treatment in the high-AIC groups for ESAS nausea (*p* = 0.027), EORTC dyspnea (*p* = 0.039), and EORTC role functioning scores (*p* = 0.031); with borderline significance for EORTC cognitive functioning (*p* = 0.061). Within-group improvement was found in the high-AIC group for ESAS sleep (*p* = 0.008) and EORTC constipation (*p* = 0.023); with borderline significance for improvement in the low-AIC group for EORTC physical (*p* = 0.082) and social functioning (*p* = 0.061).

**Table 3:** Comparison of QoL-related concerns between initial and 6-week follow-up assessment among young adult patients with cancer, comparing high- to low-AIC groups

Mean score ± SD (median)	High AIC *n* = 137		Low-AIC *n* = 43		*P* values*
	Initial visit	Follow-up visit	Initial visit	Follow-up visit	
*Edmonton Symptom Assessment Scale (ESAS)*					
Pain	4.55 ± 3.15 5 (2;7)	4.17 ± 3.11 4 (1.5;7)	3.72 ± 3.2 3 (0; 6)	4.58 ± 3.47 5 (0; 8)	P^1=^0.105 P^2^ = 0.163 P^3^=0.199 P^4^ = 0.098
Fatigue	5.99 ± 2.72 6 (4; 8)	5.58 ± 2.93 6 (3; 8)	5.53 ± 3.55 6 (2; 8)	5.63 ± 3.49 6 (2; 8)	P^1=^0.622 P^2^ = 0.151 P^3^=0.763 P^4^ = 0.349
Nausea	3.07 ± 2.99 2 (0; 6)	2.28 ± 2.85 1 (0; 5)	2.77 ± 3.39 1 (0; 5)	3.67 ± 3.93 2 (0; 8)	P^1=^0.292 P^2^ = 0.013 P^3^=0.201 P^4^ = 0.027
Depression	3.07 ± 2.96 3 (0; 5)	2.89 ± 3.04 2 (0; 5)	3.51 ± 3.19 3 (0; 6)	3.58 ± 3.68 3 (0; 7)	P^1=^0.421 P^2^ = 0.420 P^3^=0.866 P^4^ = 0.950
Anxiety	4.18 ± 3.30 4 (1; 7)	3.31 ± 3.28 3 (0; 6)	4.56 ± 3.51 5 (1; 8)	3.72 ± 3.65 3 (0; 7)	P^1=^0.578 P^2^ = 0.001 P^3^=0.129 P^4^ = 0.586
Drowsiness	5.37 ± 2.96 5 (3.5; 8)	4.80 ± 3.12 5 (2; 7)	4.93 ± 3.49 5 (2; 8)	4.93 ± 3.51 6 (1; 8)	P^1=^0.494 P^2^ = 0.039 P^3^=0.722 P^4^ = 0.758
Breathing	2.33 ± 2.90 1 (0; 4.5)	1.59 ± 2.60 0 (0; 2)	1.33 ± 2.78 0 (0; 0)	1.16 ± 1.99 0 (0; 2)	P^1=^0.005 P^2^ = 0.004 P^3^=0.954 P^4^ = 0.099
Appetite	4.04 ± 3.08 5 (0; 6)	3.88 ± 3.16 4 (1; 6)	4.02 ± 3.79 4 (0; 7)	4.58 ± 3.51 5 (0; 8)	P^1=^0.889 P^2^ = 0.281 P^3^=0.610 P^4^ = 0.435
Sleep	5.15 ± 3.07 5 (3; 8)	4.35 ± 2.90 5 (2; 7)	5.02 ± 3.46 5 (2; 8)	5.05 ± 3.29 5 (2; 8)	P^1=^0.815 P^2^ = 0.008 P^3^=0.763 P^4^ = 0.374
Well-being	5.77 ± 2.53 6 (4; 8)	5.01 ± 2.54 5 (3; 7)	5.47 ± 3.14 5 (3; 8)	6.12 ± 3.04 7 (4; 8)	P^1=^0.573 P^2^ = 0.001 P^3^=0.240 P^4^ = 0.046
*EORTC QLQ-C30 functional scales*					
Physical functioning	58.7 ± 26.3 60 (40; 80)	57.6 ± 26.3 60 (33.3;80)	56.4 ± 26.3 53 (33; 80)	50.4 ± 27.1 47 (27; 70)	P^1^=0.627 P^2^ = 0.901 P^3^=0.082 P^4^ = 0.159
Cognitive functioning	61.7 ± 29.5 67 (33; 83)	60.6 ± 30.9 67 (33; 83)	66.3 ± 30.2 67 (50; 92)	57.3 ± 40.0 50 (33; 92)	P^1^=0.321 P^2^ = 0.986 P^3^=0.013 P^4^ = 0.061
Role functioning	39.7 ± 34.1 33 (0; 67)	41.9 ± 31.6 33 (17; 67)	40.2 ± 35.4 33 (0; 67)	29.7 ± 32.4 17 (0; 50)	P^1^=0.971 P^2^ = 0.387 P^3^=0.045 P^4^ = 0.031
Social functioning	44.6 ± 34.7 33 (17; 75)	43.2 ± 33.3 50 (8; 67)	39.6 ± 35.3 33 (0; 100)	29.2 ± 34.3 17 (0; 100)	P^1^=0.413 P^2^ = 0.835 P^3^=0.061 P^4^ = 0.133
Emotional functioning	49.9 ± 27.8 50 (33,75)	53.0 ± 31.9 50 (25; 75)	50.7 ± 34.0 62 (12, 75)	51.3 ± 35.5 62 (8; 81)	P^1^=0.12 P^2^ = 0.329 P^3^=0.846 P^4^ = 0.20
*EORTC QLQ-C30 symptoms scales*					
Pain	53.2 ± 34.7 50 (17;83)	52.5 ± 34.3 50 (17 83)	50.8 ± 37.8 50 (17; 83)	52.4 ± 38.1 66 (17 83)	P^1^=0.716 P^2^ = 0.600 P^3^=0.711 P^4^ = 0.864
Fatigue	74.5 ± 29.4 89 (56;100)	72.4 ± 24.9 78 (56; 100)	71.0 ± 31.8 89 (33; 100)	74.5 ± 27.1 78 (56; 100)	P^1^=0.538 P^2^ = 0.279 P^3^=0.339 P^4^ = 0.109
Nausea	24.0 ± 24.3 17 (0; 50)	20.5 ± 22.4 17 (0; 33)	22.8 ± 25.7 17 (0; 33)	25.6 ± 27.9 17 (0; 50)	P^1^=0.656 P^2^ = 0.101 P^3^=0.644 P^4^ = 0.230
Constipation	32.3 ± 38.3 33 (0; 67)	24.1 ± 34.9 0 (0; 33)	22.5 ± 30.6 0 (0; 33)	21.7 ± 33.4 0 (0; 33)	P^1^=0.236 P^2^ = 0.023 P^3^=0.764 P^4^ = 0.345
Dyspnea	23.9 ± 31.3 0 (0; 33)	19.6 ± 28.9 0 (0; 33)	16.3 ± 29.9 0 (0; 33)	21.1 ± 30.5 0 (0; 33)	P^1^=0.100 P^2^ = 0.157 P^3^=0.206 P^4^ = 0.039
Appetite	43.6 ± 38.5 33 (0; 67)	43.3 ± 44.2 33 (0; 67)	45.8 ± 39.0 33 (0; 92)	47.5 ± 40.6 33 (0; 100)	P^1^=0.730 P^2^ = 0.511 P^3^=0.689 P^4^ = 0.552
Sleep	57.5 ± 38.4 67 (33; 100)	55.7 ± 37.4 67 (33,100)	54.2 ± 38.2 67 (8; 100)	56.7 ± 38.6 67 (33; 100)	P^1^=0.600 P^2^ = 0.681 P^3^=0.738 P^4^ = 0.522
Global health status/QoL	40.6 ± 26.8 38 (17; 58)	41.8 ± 23.5 46 (25,58)	41.0 ± 29.7 46 (17; 65)	35.4 ± 28.7 33(8; 58)	P^1^=0.839 P^2^ = 0.634 P^3^=0.307 P^4^ = 0.265
*MYCAW concerns*					
Well-being	4.0 ± 1.5 4 (3; 5)	3.4 ± 1.5 3 (2; 4)	3.8 ± 1.85 4 (3; 5)	4.1 ± 1.6 4 (3; 5)	P^1^=0.957 P^2^ = 0.002 P^3^=0.005 P^4^ = 0.008

AIC, adherence to integrative care.

^¥^ Edmonton Symptom Assessment Scale (ESAS) is a scale from 0 (lowest severity) to 10 (greatest severity).

^**#**^ EORTC QLQ-C30 (European Organization for Research and Treatment of Cancer Quality of Life Questionnaire) functioning scales has scores ranging from 0 (greatest severity) to 100 (lowest severity), while symptom scales ranging from 0 (lowest severity) to 100 (greatest severity).

^**Ω**^MYCAW, Measure Yourself Concerns and Well-being questionnaire scores the two most significant concerns ranging from 0 (not bothering me at all) to 6 (bothers me greatly).

* P values are presented concerning the following comparisons between the study groups:

P^1^= compared high vs. low-AIC group scores in initial visit (baseline scores);

P^2^= within high-AIC group score changes from initial to follow-up visit;

P^3^= within low-AIC group score changes from initial to follow-up visit;

P^4^= between high vs. low-AIC group changes from initial to follow-up visit.

### QoL changes in young vs. older patients

Table 4 presents the findings of a sub-analysis of the high-AIC group, comparing baseline-to-6-week QoL assessment between young (aged ≤ 39 years) and older (aged 40–45 years) patients. However, no between-group differences were found, with within-group improvement reported in the older subgroup for wellbeing scales on ESAS (*P* = 0.006) and MYCAW (*p* < 0.001); and for ESAS fatigue (*p* = 0.033), anxiety (*p* = 0.001) and breathing (*p* = 0.016). At the same time, ESAS sleep significantly improved (*p* = 0.019) in the young subgroup of high-AIC patients.

**Table 4:** Sub-analysis of QoL-related concerns between initial and 6-week follow-up assessment in young (18–39 years) vs. older (40–45 years) patients with cancer

Mean score ± SD (median)	Age 18–39 High AIC *n* = 65		Age 40–45 High-AIC *n* = 72		*P* values*
	Initial visit	Follow-up visit	Initial visit	Follow-up visit	
*Edmonton Symptom Assessment Scale (ESAS)*					
Pain	4.12 ± 3.1 5 (1;7)	3.85 ± 3.1 3 (1;6)	4.94 ± 3.1 5 (2; 7)	4.46 ± 3.15 4 (2, 7)	P^1=^0.131 P^2^ = 0.555 P^3^=0.153 P^4^ = 0.437
Fatigue	5.42 ± 2.94 5 (3; 8)	5.45 ± 2.78 6 (3; 8)	6.51 ± 2.42 7 (5; 8)	5.69 ± 3.07 6 (3; 8)	P^1=^0.034 P^2^ = 0.826 P^3^=0.033 P^4^ = 0.163
Nausea	2.75 ± 2.93 2 (0; 5)	1.94 ± 2.74 0 (0; 4)	3.36 ± 3.03 3 (0; 6)	2.60 ± 2.94 2 (0; 5)	P^1=^0.252 P^2^ = 0.065 P^3^=0.093 P^4^ = 0.887
Depression	2.83 ± 2.83 2 (0; 5)	2.60 ± 2.97 1 (0; 5)	3.28 ± 3.08 3 (0; 5)	3.15 ± 3.10 2 (0; 5)	P^1=^0.408 P^2^ = 0.435 P^3^=0.746 P^4^ = 0.687
Anxiety	3.62 ± 3.23 3 (0.5; 6)	3.03 ± 3.24 2 (0; 6)	4.73 ± 3.29 5 (2; 8)	3.56 ± 3.32 3 (0; 6)	P^1=^0.051 P^2^ = 0.162 P^3^=0.001 P^4^ = 0.335
Drowsiness	5.02 ± 3.16 5 (2; 7.5)	4.48 ± 3.16 5 (2; 7)	5.69 ± 2.75 6 (4; 8)	5.10 ± 3.07 5 (3; 8)	P^1=^0.251 P^2^ = 0.234 P^3^=0.074 P^4^ = 0.995
Breathing	2.31 ± 2.81 1 (0; 4)	1.63 ± 2.71 0 (0; 2)	2.35 ± 2.99 1 (0; 5)	1.56 ± 2.52 0 (0; 3)	P^1=^0.916 P^2^ = 0.094 P^3^=0.016 P^4^ = 0.957
Appetite	4.0 ± 312 5 (0; 6)	3.95 ± 3.36 4 (0; 7)	4.07 ± 3.07 4 (0.5; 7)	3.82 ± 2.99 4 (1; 6)	P^1=^0.875 P^2^ = 0.923 P^3^=0.409 P^4^ = 0.436
Sleep	5.25 ± 3.02 5 (3; 8)	4.03 ± 3.05 5 (1; 7)	5.13 ± 3.11 5 (3; 8)	4.63 ± 2.75 5 (2; 7)	P^1=^0.826 P^2^ = 0.019 P^3^=0.162 P^4^ = 0.273
Well-being	5.32 ± 2.45 5 (4; 7)	4.58 ± 2.46 5 (3; 6.5)	6.18 ± 2.53 6 (5; 8)	5.40 ± 2.56 5 (3.25; 7)	P^1=^0.056 P^2^ = 0.069 P^3^=0.006 P^4^ = 0.702
*EORTC QLQ-C30 functional scales* Initial visit: See columns CU-DX Follow-up visit: columns EQ-FT					
Physical functioning	60.7 ± 25.7 60 (40; 80)	58.9 ± 26.8 60 (40;80)	56.8 ± 26.8 60 (33; 80)	56.5 ± 26.0 60 (33; 80)	P^1^=0.427 P^2^ = 0.384 P^3^=0.562 P^4^ = 0.319
Cognitive functioning	66.1 ± 28.9 67 (50; 100)	66.1 ± 29.9 67 (50; 100)	57.7 ± 29.6 67 (33; 83)	55.6 ± 31.1 50 (33; 83)	P^1^=0.321 P^2^ = 0.826 P^3^=0.929 P^4^ = 0.061
Role functioning	44.7 ± 35.5 33 (17; 83)	47.1 ± 32.9 50 (17; 67)	35.2 ± 32.3 33 (0; 67)	37.1 ± 29.9 33 (0; 67)	P^1^=0.129 P^2^ = 0.642 P^3^=0.555 P^4^ = 0.555
Social functioning	50.0 ± 36.3 50 (17; 83)	48.1 ± 34.8 50 (17; 67)	39.6 ± 32.6 33 (0; 100)	38.6 ± 31.4 33 (0; 67)	P^1^=0.413 P^2^ = 0.483 P^3^=0.464 P^4^ = 0.133
Emotional functioning	50.8 ± 27.8 50 (33,75)	56.7 ± 32.5 62 (31; 83)	49.0 ± 27.9 50 (25, 67)	49.8 ± 31.2 46 (25; 75)	P^1^=0.740 P^2^ = 0.330 P^3^=0.675 P^4^ = 0.782
*EORTC QLQ-C30 symptoms scales* *Initial visit: See columns CU-DX* *Follow-up visit: columns EQ-FT*					
Pain	51.3 ± 34.3 50 (17;83)	48.7 ± 34.8 50 (17 83)	54.9 ± 35.3 50 (33; 100)	48.7 ± 34.8 50 (17 83)	P^1^=0.548 P^2^ = 0.443 P^3^=0.969 P^4^ = 0.644
Fatigue	71.4 ± 30.0 78 (44;100)	70.7 ± 25.3 67 (44; 100)	77.2 ± 28.7 88 (67; 100)	73.9 ± 24.6 78 (67; 100)	P^1^=0.380 P^2^ = 0.920 P^3^=0.134 P^4^ = 0.251
Nausea	22.7 ± 20.8 17 (0; 33)	17.2 ± 22.4 17 (0; 33)	25.1 ± 27.1 17 (0; 50)	23.4 ± 24.8 17 (0; 33)	P^1^=0.380 P^2^ = 0.094 P^3^=0.439 P^4^ = 0.251
Constipation	27.4 ± 34.9 0 (0; 42)	18.8 ± 31.7 0 (0; 33)	37.0 ± 40.7 33 (0; 67)	28.6 ± 37.1 0 (0; 33)	P^1^=0.229 P^2^ = 0.085 P^3^=0.131 P^4^ = 0.858
Dyspnea	25.8 ± 33.8 0 (0; 33)	22.6 ± 32.4 0 (0; 33)	22.2 ± 28.9 0 (0; 33)	16.9 ± 25.3 0 (0; 33)	P^1^=0.321 P^2^ = 0.620 P^3^=0.094 P^4^ = 0.875
Appetite	44.4 ± 39.7 33 (0; 67)	42.8 ± 36.1 33 (0; 67)	42.9 ± 37.7 33 (0; 67)	43.8 ± 50.6 33 (0; 67)	P^1^=0.833 P^2^ = 0.800 P^3^=0.500 P^4^ = 0.673
Sleep	55.0 ± 37.5 67 (33; 100)	50.8 ± 40.1 67 (0,100)	59.6 ± 39.4 67 (33 100)	60.1 ± 34.6 67 (33; 100)	P^1^=0.871 P^2^ = 0.488 P^3^=0.937 P^4^ = 0.673
Global health status/QoL	45.2 ± 27.1 42 (25; 67)	42.3 ± 24.2 50 (33,67)	36.4 ± 25.9 33 (17; 50)	38.6 ± 22.5 38(17; 50)	P^1^=0.724 P^2^ = 0.634 P^3^=0.307 P^4^ = 0.359
*MYCAW concerns*					
Well-being	3.6 ± 1.6 4 (3; 5)	3.2 ± 1.5 3 (2; 4)	4.3 ± 1.27 4.5 (3; 5)	3.5 ± 1.6 4 (2; 5)	P^1^=0.014 P^2^ = 0.096 P^3^<0.001 P^4^ = 0.151

AIC, adherence to integrative care.

^¥^ Edmonton Symptom Assessment Scale (ESAS) is a scale from 0 (lowest severity) to 10 (greatest severity).

^**#**^ EORTC QLQ-C30 (European Organization for Research and Treatment of Cancer Quality of Life Questionnaire) functioning scales has scores ranging from 0 (greatest severity) to 100 (lowest severity), while symptom scales ranging from 0 (lowest severity) to 100 (greatest severity).

^**Ω**^MYCAW, Measure Yourself Concerns and Well-being questionnaire scores the two most significant concerns ranging from 0 (not bothering me at all) to 6 (bothers me greatly).

* P values are presented concerning the following comparisons between the study groups:

P^1^= compared young vs. older adults group scores in initial visit (baseline scores);

P^2^= within young adults group score changes from initial to follow-up visit;

P^3^= within older adults group score changes from initial to follow-up visit;

P^4^= between young vs. older adults group changes from initial to follow-up visit.

### Safety-related issues

No major adverse events were attributed by patients to the IO intervention in both groups of patients. However, local and mild adverse effects were reported in a few cases following acupuncture, though these resolved and did not require cessation of the treatment.

## Discussion

This pragmatic and prospective study examined the impact of a 6-week IO treatment program on young adult patients with cancer who were undergoing active oncology treatment, within a real-world clinical setting as part of palliative and supportive care. Patients who were highly adherent to the program reported significantly improved wellbeing, more significantly among the older subgroup of high-AIC patients. Between-group analysis showed a more significant improvement in the high-AIC group for general wellbeing (ESAS and MYCAW); nausea and dyspnea (ESAS); and role functioning (EORTC). Within-group analysis of the high-AIC group showed improved scores on ESAS sleep and EORTC constipation. While these findings suggest an overall greater beneficial effect for QoL-related concerns in the high- vs. low-AIC young adult group of patients, they are less significant than what has been shown in pragmatic studies of the IO setting of older adults. For example, in the present study no effect of the IO intervention was observed on ESAS and EORTC fatigue, pain, and appetite; or on EORTC physical and emotional functioning scales. This contrasts with the findings of studies of adult patients, which found a significantly more pronounced effect for these concerns in high-AIC patients [22–24].

The present study also showed that the IO program was less effective in the young subgroup of highly adherent patients (Table 4), this for general wellbeing (ESAS and MYCAW) and ESAS scores for fatigue, anxiety, and dyspnea. It is possible that this discrepancy reflects the greater vulnerability of young patients with cancer, a population with a greater complexity of bio-psycho-social factors. These include delays in diagnosis; disrupted careers; intimacy issues, including sexuality-related concerns [25]; dignity-related distress [26]; religious/spiritual challenges [27]; a high fear of cancer recurrence [28]; challenges in coping with family responsibilities during treatment; and the risk for a negative impact on informal family caregivers [29, 30]. In Japan, Okamura et al. reported that more than 70% of young adult cancer patients reported unmet supportive care needs [31]. In a study exploring QoL in colorectal cancer survivors, Oswald et al. reported that young adults (age 18–39 years) reported worse social function on EORTC, as well as clinically meaningful social, role, and physical dysfunction, when compared to older patients [32].

A number of interventions have been shown to be effective in addresses QoL-related concerns reported by young adult patients with cancer. An RCT examining a psychosocial resilience coaching program for AYAs with advanced cancer found improved resilience and hope, though with no immediate effect on QoL [33]. Atkinson et al. found that a structured exercise intervention initiated after the completion of acute cancer treatment among AYAs improved VO_2peak_, though here too no significant beneficial effect was found for patient QoL or fatigue [34]. At the same time, a systematic review concluded that exercise is effective in managing cancer-related fatigue among AYAs [35]. A number of easily implemented interventions have been developed with the goal of addressing QoL-related concerns in this unique patient population. These include a smartphone application (App) which uses a symptom tracker and a social community platform [36]. Lopez et al. reported that physical IO interventions, such as yoga and Pilates, are of greater interest to the AYA population [37]. Still, there is has been limited research published on the impact of IO programs on AYA patients with cancer, such as a pilot study which examined the effect of a mindfulness-based intervention on emotional distress on these patients [38].

The present study suggests that the IO model is feasible for young adult patients undergoing cancer treatment. The study used a pragmatic research design, focusing on subjective patient reported outcomes, highlighting the need to further explore this model within the broader context of addressing unmet needs in this unique population of young adults. In the IO model presented here, the HCP plays a pivotal role in both the referral of the patient to the IP consultation; and in the ongoing communication with the IP following the consultation, providing input to adapt and adjust IO treatment goals and implementation. However, we suggest that the extent of integration should be further developed. The involvement of the patient’s HCP in the IO process of care should begin in the pre-hablitation stage of treatment, immediately following diagnosis and before active oncology treatment has begun (e.g., collaboration with the oncology surgeon and/or the gynecologist in the fertility clinic), as well as during and throughout the IO treatment process, including in outcome assessment.

The IO model of care needs to take into consideration the patient’s informal caregiver, who should be seen as an independent ‘client’ or ‘case’ who can also benefit from these treatments. The parallel treatment of the caregiver needs to take place in an ongoing collaboration and communication with the patient’s oncologist, nurse-oncologist, psycho-oncologist, and palliative care practitioner. The IO team needs to facilitate a comprehensive process of integration in the care of young adult patients, beyond the simple context of providing acupuncture and other IO modalities, working in close collaboration with the HCP acting as the patient’s case manager. In this way, a synergistic interaction can take place with all who are involved, directly or indirectly, in the oncology, palliative, and paramedical care of the young adult patient.

This study has a number of significant methodological limitations which preclude reaching any conclusions from the findings. The first and most important of these is the pragmatic and non-randomized approach employed, using a preference-controlled design with patients determining their own adherence to the IO program. Despite the retrospective nature of defining high vs. low adherence at 6 weeks, the IO team monitored adherence throughout the 6-week intervention, with the goal of increasing attendance at scheduled treatment sessions. However, adherence itself may function as a proxy for unmeasured factors such as health literacy; psychological resilience; social support; informal caregiver support (including prioritization of escorting or driving the patient to treatments); cost-related limitations (e.g., travel to the oncology center); symptom burden; or prior experience and belief in integrative medicine. Notably, despite the risk for a preference bias, both high- and low-AIC groups had largely similar baseline demographic and cancer-related parameters.

In addition to these limitations, the lack of young adult-specific baseline characteristics identifying the two subgroups (presented in Table 1) preclude reaching generalizable conclusions regarding this patient population. While education, occupation, income, marital status, number of children and informal caregivers are important aspects to consider in young adult studies, assessment of other factors such as parenting status, fertility-related distress, partnership status, and dependency on caregivers may have enriched the understanding of the participants’ concerns and wellbeing. Future studies should not only include these parameters but also compare sub-groups of young adults, such as those in the age range of 18–25, to older groups. A focus on this young group of patients would address types of primary cancer which are different from those observed in the present cohort. In addition, more research is needed to explore young adult patients who were not referred to the IP consultation, or who were referred but did not attend. This would better address a potential referral bias, as well as to explore patients’ perceptions, motivation, and barriers to attending the IO consultation and treatments.

The clinical design of the IO interventions used in the study are based on the SIO-ASCO clinical guidelines, which do not specifically address the young adult patient population. More research is still needed to better understand the effects of IO in young adults, not necessarily in the context of the treatment of children and adolescents. Ideally, these studies should also include a pragmatic research design to explore the patient-tailored IO approach and the ‘dose effect’ of integrative care, based on specific IO modalities and/or adherence to weekly treatments. Combining qualitative research methodology with patient-reported outcomes may also highlight non-specific effects of the IO intervention, while considering challenges faced by these patients which may significantly affect their wellbeing. These include developmental stage along the life cycle; identity formation; autonomy; body image; fertility-related concerns; and social role-related disruption.

Another study limitation is the use of subjective patient-reported outcome study tools, as opposed to objective parameters such as monitoring adherence to the conventional oncology treatment protocol as reported in other IO studies assessing relative dose intensity (RDI) among patients treated with taxane and/or platinum-based agents [39, 40]. Healthcare utilization is another objective parameter assessed in previous IO studies suggesting fewer dispensed opioid prescriptions and lower rates of hospitalizations [41]. The lack of longitudinal follow-up beyond six weeks further constrains interpretation of the present study. Finally, the generalizability of the findings in the study setting needs to be confirmed through the examination of other IO programs, both in Israel and globally. Further research, ideally within an RCT format, should focus on specific oncology patient populations and/or age groups.

In conclusion, the present study explored the impact of an IO model of care in young adult patients with cancer undergoing active oncology treatment. High adherence to weekly IO treatments following an initial IO consultation was associated with significantly improved wellbeing at 6 weeks. At the same time, a lesser impact of the IO program was observed in a young subgroup of high-AIC patients (aged 18–39 years), in which no improvement was observed in leading QoL-related concerns such as pain, fatigue, and appetite. The study findings highlight the need to design a young adult-tailored IO treatment strategy in accordance with the unique psycho, social, and spiritual-related concerns among this patient population, especially for those undergoing active oncology treatment.

## Data Availability

No datasets were generated or analysed during the current study.
